# Chronic Mesenteric Ischemia: Differential Vascularsurgical Therapy and Its Outcome in a Single-Center Observational Study

**DOI:** 10.1159/000519423

**Published:** 2021-11-29

**Authors:** Mohamed Essa, Frank Meyer, Robert Damm, Zuhir Halloul

**Affiliations:** ^a^Division of Vascular Surgery, Department of General, Abdominal, Vascular and Transplant Surgery, Otto-von-Guericke University with University Hospital, Magdeburg, Germany; ^b^Department of General, Abdominal, Vascular and Transplant Surgery, Otto-von-Guericke University with University Hospital, Magdeburg, Germany; ^c^Department of Radiology and Nuclear Medicine, Otto-von-Guericke University with University Hospital, Magdeburg, Germany

**Keywords:** Chronic mesenteric ischemia, Open reconstruction, One-/ two-vessel reconstruction, Antegrade/retrograde reconstruction

## Abstract

**Aim:**

The aim of this study was to investigate short-/long-term vascularsurgical patency and the outcome in chronic mesenteric ischemia (CMI) depending on the mesenteric revascularization technique and reflecting real-world data.

**Methods:**

This retrospective single-center observational study registered all patients who had undergone open vascularsurgical reconstruction because of CMI at a tertiary German university hospital comparing 1-versus (vs.) 2-vessel as well as antegrade versus retrograde reconstructions.

**Results:**

In total, 35 patients were enrolled (mean [± SD] age, 64 ± 13 [range, 45–83] years; sex ratio [m:f], 16:19 [46:54]) over 12 years. Three patients with symptoms of mesenteric ischemia because of rare causes (radiation-induced and median arcuate ligament syndrome) have been excluded. While 51% of patients underwent 1-vessel reconstruction, 49% underwent 2-vessel reconstruction. There was a trend of (i) more perioperative complications in the 2-vessel group (88.2% vs. 55.6%, *p* = 0.06) and (ii) higher morbidity at 1 year in the 2-vessel versus 1-vessel group (57.1% and 42.9%, respectively; *p* = 0.466), while the morbidity of the 2-vessel versus 1-vessel group at 5 years (100% vs. 33.3%) was significantly different (*p* = 0.009). The mortality was greater in the 2-vessel versus 1-vessel group as it was significantly different in the early postoperative period (31.3% vs. 0, *p* = 0.016) and at 1 year (50% vs. 0, *p* = 0.005) and 5 years (100% vs. 11%, *p* = 0.003). Regarding overall survival, the 1-vessel group showed a significant superiority above the 2-vessel group (*p* = 0.004). Actually, there was no significant difference of early postoperative morbidity comparing the retrograde and antegrade group (*p* = 0.285) as well as at 1 year and 5 years (*p* = 0.715 and *p* = 0.620, respectively). In addition, there was no significantly different postoperative mortality in antegrade versus retrograde group at each time. Specific and general complication rates were 62.9% and 57.1%, respectively, resulting in an overall morbidity of 77.1% (mortality, 20%).

**Conclusion:**

The vascular surgeon should be prepared to perform various procedures of mesenteric reconstruction to tailor the operative strategy to the specific needs of the individual patient.

## Introduction

Mesenteric ischemia is a rare but severe disease, which is encountered during vascular surgery practice. This disorder is either acute or chronic based on the acuity and duration of symptoms. Chronic mesenteric ischemia (CMI) is a chronic and insidious process, which usually progresses over several months. Those patients usually would have undergone an extensive diagnostic workup for other suspected etiologies [1].

The first description of mesenteric vascular occlusion was attributed to the pathologist Antonio Benivieni from Florence in the later part of the fifteenth century [2]. CMI secondary to arterial insufficiency was first recognized and described by Chienne [3] in 1868 followed by Councilman [4] in 1894 with the anatomical description of the celiac trunk and superior mesenteric artery (SMA) occlusions. CMI was first described as “abdominal angina” in 1918 by Goodman [5]. In 1958, the first successful open repair for CMI was performed by Shaw and Maynard [6], and they reported 2 cases successfully treated using thromboendarterectomy. Technically, more successful procedures, such as Dacron bypass grafting from the infrarenal aorta to the SMA, were described in 1962 by Morris et al. [7]. Moreover, antegrade aortovisceral bypass and transaortic visceral thromboendarterectomy were described in 1966 by Stoney et al. [8]. The French advanced a new technique to revascularize the SMA, often in association with a reconstruction of the infrarenal aorta using retrograde bypass in a left retroperitoneal C-shaped route behind the renal pedicle to revascularize the SMA in an antegrade manner. It is often called “French bypass” [9].

According to some estimations, up to 95% of cases of CMI are due to atherosclerosis. Nonatherosclerotic causes account for 5–10% of all cases of CMI [10], such as aortic dissection, retroperitoneal fibrosis, vasculitis, and postradiation exposure [11]. The majority of patients (75%) are smokers. About one-third of patients have hypertension and hyperlipidemia. Approximately 10% of patients are diabetic [12]. Open surgical treatment using bypass was considered the gold standard of treatment in the past. However, the endovascular treatment, consisting of percutaneous transluminal angioplasty and stenting, has emerged recently as an alternative treatment modality for CMI [13]. Several studies recommend single-vessel reconstruction using the autologous vein and a retrograde approach with bypass grafts originating from the infrarenal aorta [14]. The aim was to review more than 12-years' experience of open vascularsurgical treatment for patients with CMI from a single institution, in particular, the short- and long-term vascularsurgical outcome depending on the mesenteric revascularization technique using 1 vessel or 2 vessels and either antegrade or retrograde flow direction of reconstruction as well as reflecting real-world data.

## Methods

All consecutive patients who had undergone an elective open vascular reconstruction of CMI by the vascularsurgical team at a (tertiary) university center of (endo-)vascular surgery in Germany over a defined period of time were:

1. enrolled in this systematic retrospective uni-center observational study for quality assurance to reflect daily vascularsurgical practice as a contribution to research on clinical care (design), and
2. documented in a clinical database.

Any form of CMI was considered (inclusion criteria). Nonocclusive mesenteric ischemia, mesenteric venous occlusion, acute mesenteric ischemia, or visceral artery reconstructions for aneurysmatic disease and patients with mechanical compression of the celiac artery by the median arcuate ligament were excluded (exclusion criteria).

**Fig. 1:**
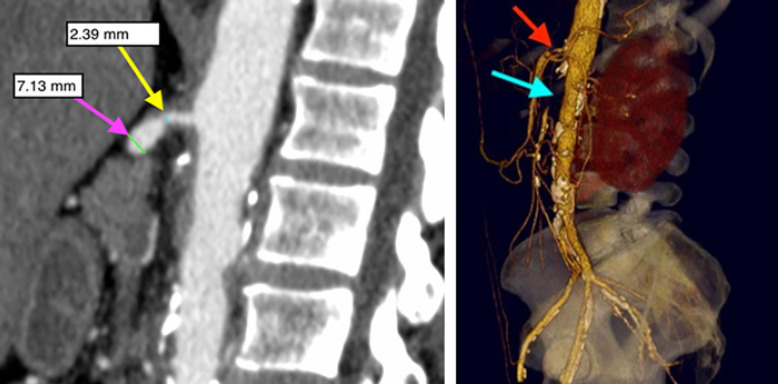
Contrast-enhanced multi-slice CT showing a CT-slice of a sagittal reconstruction in a patient with high-grade stenosis of the CA at its origin (>70% reduction of arterial diameter) and occlusion of the proximal SMA (left) and a 3-D reconstruction of the same patient (right) − red arrow represents the CA-stenosis, and light blue arrow represents the SMA-occlusion (from the Department of Radiology and Nuclear Medicine, University Hospital of Magdeburg [Germany]). SMA, superior mesenteric artery.

**Fig. 2:**
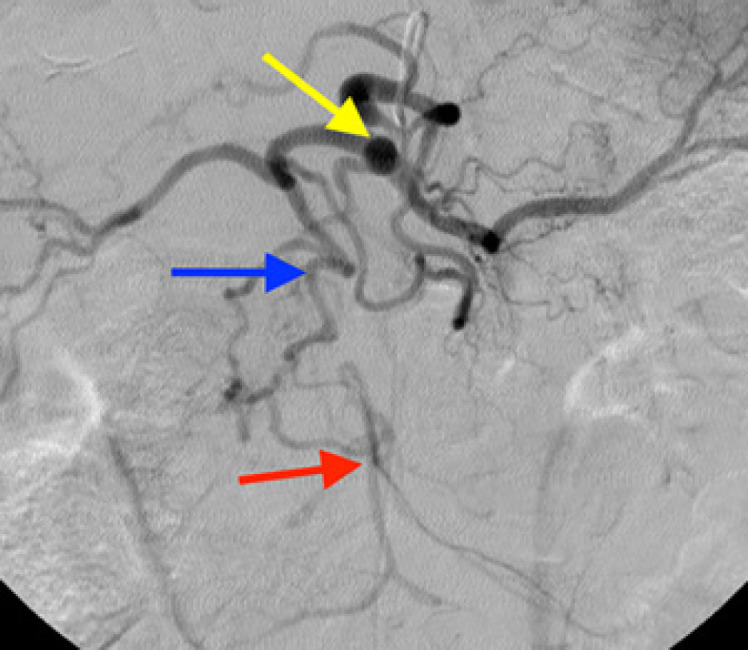
Selective angiography representing a long-standing obstruction of the SMA with well-developed collaterals through the GDA (CA: yellow arrow, GDA: blue arrow, and SMA: red arrow) (from the Department of Radiology and Nuclear Medicine, University Hospital of Magdeburg [Germany]). SMA, superior mesenteric artery.

Repairs utilizing bypass conduit originating from the supraceliac aortic or transaortic endarterectomy were considered antegrade. Inflow originating from the distal aorta or iliac artery was considered retrograde. The patients' group was followed up initially at 3 months and then once yearly. The presence or absence of change in clinical symptoms including postprandial abdominal pain, weight loss, and food fear was determined. Mesenteric duplex ultrasonography was the first diagnostic tool to consider if there was a clinical suspicion of recurrent symptoms. Further investigations including CTA, MRA, and DSA are considered as shown in Figures 1 and 2.

Nonpersonal-specific anonymized data had been gathered and used in the current study. The follow-up endpoint was either loss from follow-up or death. A Medline/PubMed search from early 1990 through June 2018 was conducted, depending upon search criteria of CMI, risk factors, bypass, preoperative, intraoperative, and open reconstruction. This search yielded around 80 articles meeting the authors' primary interest. Every study or article presenting any form of at least 1 risk factor correlation with the clinical outcome after open mesenteric reconstruction of CMI was included as illustrated in online supplementary Table 1; see www.karger.com/doi/10.1159/000519423 for all online supplementary materials.

The diagnosis of CMI is based primarily on clinical symptoms and supported by imaging findings, following the exclusion of other potential intestinal disorders. CT accurately demonstrates calcified and noncalcified plaque causing arterial stenosis or occlusion, typically in the proximal CA and SMA [15].

All 3 mesenteric arteries “CA, SMA, and IMA” were assessed either as obstructed or stenotic. High-grade stenosis of visceral arteries was defined as decreased vessel diameter of >70% [16].

Several techniques were used to reconstruct the diseased visceral arteries either as 1 or 2 mesenteric artery repairs. The reconstruction was performed either in an antegrade or retrograde fashion using usually a transperitoneal and sometimes a retroperitoneal approach. Graft size was based on the diameter of the CA and SMA. If reconstruction of the CA and SMA was planned, a preformed Y-graft was used. If the entire celiac trunk was occluded or diseased, the preferred target artery was the common hepatic artery [17]. A mesenteric bypass was used using a venous graft in some cases if appropriate venous material was available either mesenteric vein or more commonly great saphenous vein, and those patients had elevated risk for wound infection. A retrograde reconstruction was considered for patients with high risk for cardiovascular events during aortic cross-clamping and for those with extensive calcification of the supraceliac aorta. The retrograde bypass originated either from the iliac artery, the infrarenal aorta, or even aortic prosthesis directly or in a C-shaped configuration. Either a polyester (Silver Graft®, B. Braun Deutschland GmbH & Co. KK, Berlin, Germany), ePTFE (PROPATEN®, W.L. Gore & Associates, Newark, DE, USA), or venous graft was chosen for the bypass. Mesenteric endarterectomy was done in a few cases with closure of the arteriotomy using a xenogenic patch (XenoSure®, LeMaitre Vascular, Burlington, VT, USA).

Primary patency was defined as symptom relief and was considered the sole determinant of successful therapy. Clinical patency was regarded as asymptomatic patients postoperatively, independent of graft patency, which might be proven radiologically to be occluded. Secondary patency was defined as a symptom relief after secondary intervention following the primary operative procedure in the follow-up period. The possible perioperative, in particular, postoperative complications were considered part of peri-/postoperative morbidity, which occurred from the first postoperative period until the discharge of patients.

### Statistical Methods

The statistical evaluation was carried out using the software SPSS Statistics, version 24.0 (SPSS Inc., Chicago, IL, USA). Nonparametric tests were performed to determine whether there was a significant difference between the findings and the individual variables. Survival and patency rates were calculated using the Kaplan-Meier assessment method. Univariate statistical comparisons using contingency table analysis (Pearson's χ^2^ test) were made between each of the directionality of reconstruction (antegrade or retrograde) and the number of reconstructed arteries (either 1 or 2 arteries), regarding the development of complications postoperatively. A statistically significant difference was ascribed to a *p* value of <0.05.

## Results

From 2005 to 2018, 45 patients were identified who had undergone mesenteric revascularization for CMI. Eight patients who had received revascularization of acute on top of CMI and 4 patients who had undergone mesenteric reconstruction because of another cause such as combined visceral arterial aneurysms were excluded. Thus, 33 patients remained for final analysis. Two patients underwent a second mesenteric revascularization as a result of recurrent symptoms and graft failure, so there was a total procedure's number of *n* = 35.

The mesenteric reconstruction was performed slightly more common in women (*n* = 19, 54%) than men (*n* = 16, 46%). The mean age of patients (mean ± SD) was 65 ± 11.87 (range, 45–83) years.

Atherosclerosis was the cause of CMI. The most frequently reported symptoms were postprandial abdominal pain in all patients and >10% with body weight loss during the last 6 months (*n* = 30 patients, 86%), with a mean ± SD of BMI as 20.97 ± 4.28 kg/m^2^. Only 2 patients presented with gastrointestinal bleeding. The distribution of preoperative risk factors among males and females is listed in online supplementary Table 2.

In detail, 97% of patients had affection of the SMA. The anatomical distribution of pathologies of mesenteric arteries is listed in Table 1. The material used for reconstruction included polyester, PTFE, venous, and biological grafts as well as combination of several materials.

**Fig. 3:**
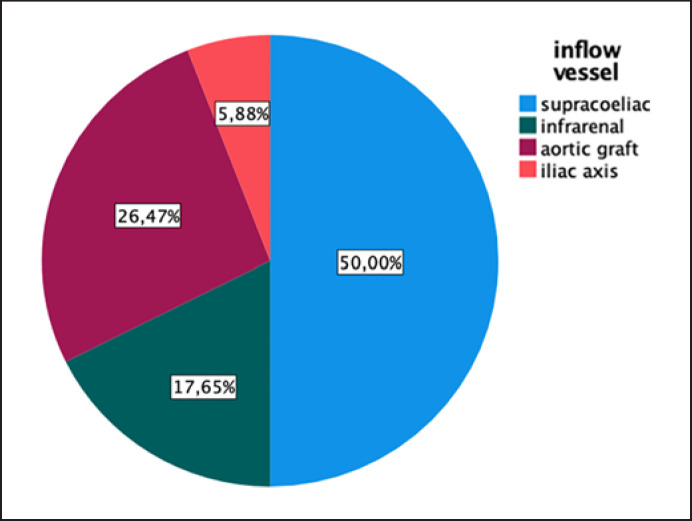
Frequency of various inflow feeding arteries used for reconstruction.

**Fig. 4:**
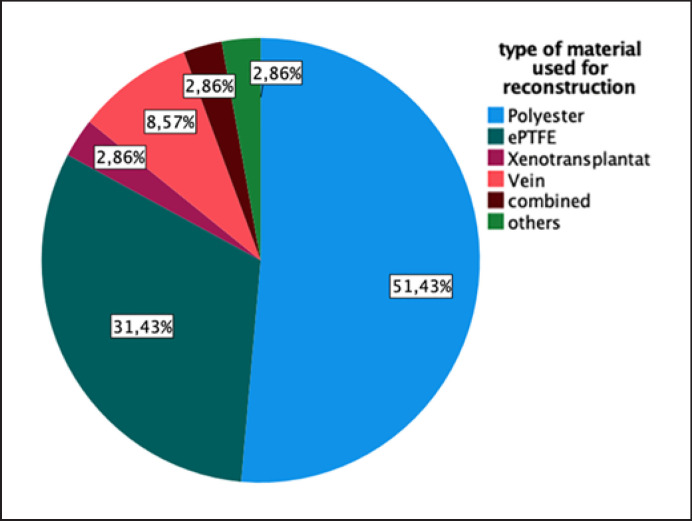
Distribution according to the type of material used for mesenteric reconstruction (polyester/dacron, ePTFE, vein graft, biologic/xenogenic material, combined reconstruction, and others/endarterectomy).

Various inflow feeding vessels used in the reconstruction are demonstrated in Figure 3. Different materials for the reconstruction are used as shown in Figure 4.

Eighteen patients (51.4%) underwent 1-vessel reconstruction, whereas 17 patients (48.6%) underwent 2-vessel reconstruction. There was no combined approach of an image-guided intervention and open vascular surgery. The mean (± SD) time for cross-clamping of the aorta was 13.8 ± 9.0 min and 9.9 ± 10.2 min for cross-clamping of the supraceliac aorta, respectively.

The mean length of operation (± SD) was 196 ± 66.7 min ranging from 100 to 350 min. The mean hospital stay was 36.6 days, while the mean stay on the intensive care unit was 17.3 days.

During the postoperative period, the primary patency rate was 82.9%, whereas the clinical patency was 91.4%, and the secondary patency rate was 85.7%. At 1 year, the primary and clinical patency rate was 95.5%. After 5 years, the primary and clinical patency rate was reported to be 77.1% of the patients' group (*n* = 27/35), whereas 8 patients were lost to follow-up. A superficial surgical site infection occurred in 4 patients (11.4%) who were successfully treated with conservative wound management, whereas a deep surgical site infection including graft infection was reported in only 2 patients (5.7%), which has been treated surgically. Cardiovascular events occurred in only 3/35 patients (8.6%); respiratory complications including pneumonia, pleural effusion, and respiratory decompensation occurred in 42.9% of patients (*n* = 15/35). There was an increase of the serum creatinine level in 5/35 patients (14.3%); self-limited hepatobiliary complications including self-limiting pancreatitis and acalculous cholecystitis developed in 4/35 patients (11.4%).

Additional complications included cerebrovascular events, which occurred in 8 patients (22.9%). Postoperative surgical site bleeding occurred in 40% of patients (*n* = 14/35); of those, 9 patients required reoperation to stop the bleeding or to remove the intra-abdominal hematoma. Peripheral vascular ischemia in the lower extremities developed in only 2 patients (5.7%). Portal vein thrombosis occurred in 1 patient (2.9%). Pulmonary embolism developed in 1 patient (2.9%). Twenty percent of patients (*n* = 7/35) developed urinary tract infection, which was successfully treated with conservative management using antibiotics. Upper gastrointestinal bleeding occurred in 8.6% (*n* = 3/35).

Taken together, the specific complication rate was 62.9%, whereas the general complication rate was 57.1%, resulting in an overall morbidity of 77.1%. The overall mortality was 20%.

There was no significant difference in the postoperative morbidity in the early postoperative period, comparing retrograde with antegrade group (*p* = 0.285). The 2-vessel group had a trend of higher morbidity (*p* = 0.06) versus 1-vessel group.

At 1 year postoperatively, both patients who had undergone retrograde versus antegrade and 2-vessel versus 1-vessel reconstruction showed no significant difference regarding morbidity (*p* = 0.715 and *p* = 0.466, respectively). At 5-years follow-up, in patients with retrograde versus antegrade reconstruction, there was no different morbidity (*p* = 0.62); however, the 2-vessel group had a significantly higher morbidity (*p* = 0.009) than that of the 1-vessel group. Consistently, postoperative mortality was significantly greater in the 2-vessel group in the early postoperative period (*p* = 0.016), at 1 year (*p* = 0.005), and at 5 years postoperatively (*p* = 0.003), respectively.

The preoperative comorbidities and anatomical factors were analyzed with univariate analysis using the χ^2^ test to calculate the correlation with morbidity and mortality in the early postoperative period, after 1 year and after 5 years as shown in Table 2. The postoperative outcome including primary patency and postoperative complications in relation to directionality of reconstruction and number of treated vessels and its statistical value is listed in Table 3.

**Fig. 5:**
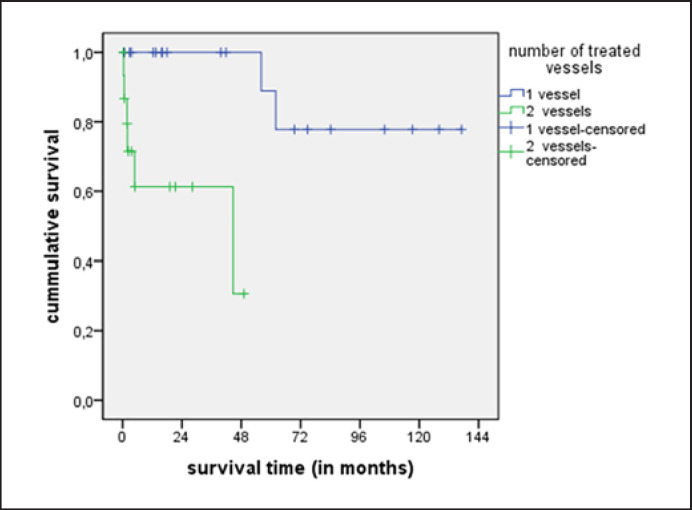
Cumulative survival comparing 1-vessel and 2-vessel groups.

Regarding the survival in relation to the number of reconstructed vessels, there is a statistically significant longer cumulative survival in the 1-vessel group (*p* = 0.001) than that in the 2-vessel group as shown in Figure 5. There was no significant difference regarding the survival of patients who had undergone antegrade versus retrograde reconstruction.

## Discussion

Because of the rarity of the disease and the nature of this single-center study, the authors confronted here the data from the current study with previous reports; there are some similarities as well as differences as listed in Table 4. The revascularization of the CA by a retrograde bypass, which terminates at the hepatic artery, was described by several authors in many instances [18].

Courbier et al. [19] attributed substantial importance to the hepatic artery. He performed an end-to-end anastomosis on it or reimplanted it, after transection, to the side of an aortomesenteric graft.

In the current study, the common hepatic artery was revascularized in 1 case via the left common iliac artery using venous conduit bypass. This avoided the necessity of prolonged cross-clamping of the supraceliac aorta. However, in this report, a clamping time of only 9.8 ± 10.2 min was observed.

In this report, no case with pathological involvement of the SMA alone was included, although other authors have described patients with CMI in the presence of pathological changes at only 1 single mesenteric vessel, usually the SMA [20, 21] or rarely the CA [8]. It is generally agreed that evidence of severe occlusive disease that involves at least 2 of the 3 mesenteric vessels is necessary to support the diagnosis of CMI [22].

The current analysis demonstrated that 30% of patients experienced at least 1 major complication during their hospitalization. Table 5 summarizes the clinical outcome of the current study in comparison to other studies in the literature. A low recurrence rate of CMI and high 1-year patency rate of 4.5 and 95.5%, respectively, are confronted by a mortality and morbidity of the upper range, which has been reported here very honestly.

In the current study, the mesenteric vessels were reconstructed using antegrade bypass in 19 patients (54.3%) and retrograde graft implantation in 16 patients (45.7%). In a study from the Cleveland Clinic, 40% of patients underwent retrograde bypass, whereas only 29% underwent antegrade bypass; the remaining patients underwent other reconstructions including local endarterectomy with local patch angioplasty. They performed 1-vessel reconstruction in 75% of patients; the residual 25% underwent 2-vessel reconstruction [23].

A few studies reported patients with CMI who had been treated with anterograde supraceliac aortomesenteric bypass grafting through upper abdominal exposure and pancreatic displacement to expose the SMA [24]. These authors saw advantages in this technique because there was:

1. less turbulence in blood flow,
2. less bypass compression by the mesentery,
3. prolonged patency of the vessel reconstructions with better flow capabilities, and
4. easier technical handling than retrograde bypass grafting.

In addition, the arteriosclerosis is usually less manifested at the supraceliac segment of the aorta. In contrast, many authors favor the better accessible approach to the infrarenal aorta [23].

Furthermore, elderly patients and those with cachexia or severe cardiac, pulmonary, and renal dysfunction are frequently not good candidates for aortic procedures. One of the main problems in retrograde bypass grafting is bypass kinking because of the mobility of the SMA.

Retrograde prosthetic bypass grafting to the SMA was performed alone or in conjunction with aortic reconstruction in 42.4% of patients in this current study. Thus, the advantage of not necessitating dissection or cross-clamping of the supraceliac aorta was found in the current study, which is a preference advocated by other authors [18, 25, 26, 27]. The major disadvantage of this approach is that care must be taken to place the graft in a near-vertical orientation from its origin to its termination to minimize the tendency to kink when the viscera return to its normal anatomical location [28, 29].

Although there are strong proponents for antegrade bypass reconstruction, there is no statistical superiority yet, as has been shown in a randomized controlled trial because of the rarity of the disease. The antegrade orientation allows for a short segment bypass, which:

1. has no propensity to kink,
2. provides direct inline flow with low turbulence, and
3. avoids direct contact with the bowel [8, 28, 29, 30, 31, 32, 33].

However, it was found in the current study that the comparison of the antegrade versus retrograde group shows a slight trend of a higher primary patency rate in the early postoperative time frame (89.5% vs. 75.0%, *p* = 0.379). This difference was not found at longer follow-up time points, such as at 1 year (92.3% vs. 100%, respectively, *p* = 1.0) and 5 years (100% primary patency in both groups) for the remaining patients. There were no significant differences of the complication and survival rates comparing the antegrade and retrograde groups except in the major postoperative bleeding. The retrograde group had only a trend of more major postoperative bleeding probability than in the antegrade group (37.5% vs. 15.8%, *p* = 0.245).

Hollier et al. [34]found that there was a 29% recurrence rate of symptoms after revascularization of 2 of 3 involved vessels. In contrast to a single-vessel reconstruction, the recurrence rate was about 50%. Thus, they suggested that although single-vessel revascularization may relieve symptoms, the optimal long-term result can be obtained by complete revascularization of all stenotic vessels. A complete revascularization was also recommended by McAfee et al. [35].

In the current study, the benefits of complete revascularization (rather related to the 2-vessel group), however, were attempted to be obtained, confronted by a trend of more early postoperative complications (88.2% vs. 55.6%, *p* = 0.06). The mortality in the early postoperative period was higher in the 2-vessel group (31.3% vs. 0, *p* = 0.016), which was statistically significant.

Although the postoperative morbidity at 1 year was higher in the 2-vessel versus 1-vessel group (57.1% vs. 40%, respectively), it did not show any significant difference. Nevertheless, the morbidity of the 2-vessel versus 1-vessel group at 5 years (100% vs. 33%) was significantly different (*p* = 0.0094). The mortality was greater in the 2-vessel versus 1-vessel group with a statistically significance in the early postoperative period (31.38% vs. 0, *p* = 0.016) and at 1 year (50% vs. 0, *p* = 0.005) and at 5 years (100% vs. 11%, *p* = 0.003).

Tertiary referral centers have reported excellent results with open reconstructions, including a recent series from the Mayo Clinic, with a mortality of 0.9% in low-risk patients [36]. In contrast, in the study presented here, rather consecutive patients matching the inclusion and exclusion criteria (as appropriate indicated), with (partially) a remarkable number and spectrum of risk factors (as listed in online suppl. Table 1, 2) were enrolled, finally resulting in a higher mortality as honestly reported. Regarding overall survival, the 1-vessel group showed superiority above the 2-vessel group, with a significant difference (*p* = 0.001).

Implications of the study confirm that mastering a variety of surgical techniques can provide durable relief of mesenteric ischemia (symptoms) and long-term symptom-free survival. The vascular surgeon should be prepared to use all the available techniques and to tailor the operative strategy to the specific needs of the individual patient.

This study has the usual limitations of any retrospective study, which are assumed to have more bias since the study operations, data collected, data entry, and data quality assurance were not planned ahead of time. It also encompasses a relatively small number of patients. These shortcomings, however, highlight a common problem regarding the CMI: its rareness.

It is not likely that a single center can gather a large enough case series during a relatively short time period in order to provide substantial data from a prospective randomized study. Finally, this study represents a retrospective report, and surgeons' bias and patients' conditions that affected the choice of conduit could not be satisfyingly identified.

## Conclusion

The current report represents outcomes in contemporary practice for operative treatment of CMI. Mesenteric reconstruction in case of CMI can be performed safely and effectively with an acceptable mortality. Although mortality was higher in patients with vein grafts than those with prosthetic conduit, it is believed that the patient condition at the time of operation was the primary determinate of the outcome. Bowel resection was required in some patients, indicating that patients with CMI can progress to bowel infarction. Therefore, it is critically important to revascularize patients expeditiously before the development of bowel infarction, a condition that increases the risk of operative mortality.

The use of 2-vessel reconstruction did not improve the patency of bypass and has resulted in higher complication rates. The survival rate has been reported being superior in the 1-vessel reconstruction group. Conceding the uncertainties for the number of vessels to be reconstructed and directionality of reconstruction, the vascular surgeon should currently attempt to reconstruct using the antegrade reconstruction of the most affected mesenteric artery if the anatomy is feasible. In summary, the resulting main points of the presented study are:

1. There is a great importance of early reconstruction of symptomatic CMI to avoid bowel infarction, and
2. Reconstruction of 1 vessel in CMI is more favorable.

## Statement of Ethics

Data generation, documentation, and evaluation were performed according to prerequisites of data protection law of the German district Saxony-Anhalt and according to the federal law. The study was performed according to the requirements of the “Declaration of Helsinki for Biomedical Research from 1964” by the “World's Medical Association” and its further amendments as well as the policy of the institutional Ethics Committee. With regard to the study concept, it can be stated that a (potential) danger for study participants can be definitely excluded. The statement of an Ethics Committee is not required since, in particular, only patient-associated data were registered independently of the (specific interests of single) patients. There is no imponderable risk or side effect for the patient as it may become possible in the use of any medication. Furthermore, the register with patient-associated data has been led according to the requirements of the German “Landes-und Bundesdatenschutzgesetz”; and then, data were evaluated anonymously without any possible interference to an individual patient. In addition, data have been monitored, validated, and evaluated at an institution close to a university hospital, a tertiary center, and with an associated University Medical School; one of their basic tasks is (according to the “Hochschulmedizingesetz” in Germany) to perform clinical research in addition to clinical care for patients. Last but not the least, leading such register of patient data can be considered close to epidemiological studies, which do not need any statement of an Ethics Committee as well as according to the “Allgemeinen Vertragsbedingungen” of the local contract between the physician and the patient (”Arzt-Patienten-Vertrag”) in its current version from 2006, article [§] 16, paragraph [subparagraph] 4, it is not permitted to evaluate anonymous data. With regard to the availability of data and materials, each patient signed informed consent form prior to (i) surgery including appropriate explanation of the surgical intervention and potential complications and (ii) generation and documentation of data in the patient data registry.

## Conflict of Interest Statement

M.E., F.M., R.D., and Z.H. declare that no potential conflicts of interest exist. This includes, but is not limited to, any financial relationship with regard to the research presented. There is no financial interest/arrangement with 1 or more organizations that could be perceived as a real or apparent conflict of interest in the context of the subject of this article for each of the authors.

## Funding Sources

This study did not receive any specific grant from funding agencies in the public, commercial, or nonprofit organization sectors.

## Author Contributions

M.E. contributed to conceptualization, data curation, formal analysis, investigation, and methodology, project administration, resources, supervision, validation, visualization, and roles/writing − original draft. F.M. contributed to data curation, formal analysis, resources, software, visualization, and writing − review and editing. R.D. contributed to data curation, formal analysis, project administration, resources, and writing − review and editing. Z.H. contributed to conceptualization, formal analysis, investigation, methodology, project administration, validation, and writing − review and editing. All the authors have read and approved the manuscript.

## Data Availability Statement

The data that support the findings of this study are not publicly available due to their containing information that could compromise the privacy of research participants but are available from corresponding author (F.M.) and first author (M.E.) upon reasonable request.

## Supplementary Material

Supplementary data

Supplementary data

## Figures and Tables

**Table 1:** Anatomical distribution for pathologies of celiac axis (celiac), SMA, and IMA

Occluded/stenotic	Patients, *n* (%)
Celiac/SMA/IMA	12 (31.6)
Celiac/SMA	17 (44.7)
SMA/IMA	6 (15.8)
Celiac/IMA	1 (2.6)
SMA	0
Celiac	2 (5.3)

Celiac, celiac axis; SMA, superior mesenteric artery; IMA, inferior mesenteric artery.

**Table 2:** Morbidity and mortality (early postoperatively, at 1 year and 5 years) with their correlation to preoperative risk factors

Analyzed risk factor	Morbidity/mortality		
	early postoperative *p* value	at 1 year *p* value	at 5 years *p* value
Gender	0.358/0.132	0.594/0.164	0.280/0.714
Age	0.411/0.791	0.591/0.724	0.388/0.359
Diabetes mellitus	0.652/0.196	0.930/0.139	0.829/0.438
Arterial hypertension	0.652/0.207	0.233/0.166	0.280**/0.070**
BMI	0.536/0.289	0.456/0.361	0.324/0.449
HLP	0.632/0.100	0.018*/0.054	0.040*/0.245
Preoperative TPN	0.215/0.915	0.809/0.918	0.308/0.185
History of smoking	0.173/0.475	0.583/0.504	0.197/0.333
PAD	0.020*/0.314	0.200/0.220	0.024*/0.398
Cerebrovascular disease	0.970/0.314	0.157/0.454	0.255/0.310
Renovascular disease	0.220/0.565	0.962/0.504	0.732/0.155
CHD	0.359/0.074	0.025*/0.017*	0.130/0.038*
CHF	0.103/0.648	0.254/0.436	0.412/0.268
CRF	0.096/0.416	0.886/0.353	0.088/0.464
Previous malignancy	0.191/0.925	0.025*/0.812	0.07/0.919
Previous abdominal surgery	0.874/0.523	0.901/0.558	0.952/0.310
Previous bowel surgery	0.163/0.585	0.870/0.453	0.914/0.398
Previous peripheral vascular surgery	0.489/0.380	0.318/0.139	0.070/0.038*
Previous carotid surgery	0.946/0.295	0.294/0.026*	0.412/0.268
Previous aortic surgery	**0.087**/0.482	0.739/0.362	0.308/0.185
Previous cardiac surgery	0.765/0.024*	0.060/0.017*	0.231/0.104
Previous mesenteric surgery	**0.065**/0.377	0.583/0.436	0.732/0.919

BMI, body mass index; HLP, hyperlipoproteinemia; TPN, total parenteral nutrition; PAD, peripheral arterial disease; CHD, coronary heart disease; CHF, congestive heart failure; CRF, chronic renal failure.

* Statistically significant.

**Table 3:** Postoperative outcome and complications and their correlation to both the directionality of reconstruction (antegrade vs. retrograde) and the number of reconstructed vessels (1-vessel vs. 2-vessel)

	Antegrade (*n* = 21)	Retrograde (*n* = 17)	1-vessel (*n* = 20)	2-vessel (*n* = 18)
Primary patency − early postoperative, %, *n p* value	90.5, 19	90.5, 19	85, 17	77.8, 14
	0.207		0.687	
Primary patency − at 1 year, %, *n p* value	92.3, 12	100, 9	92.9, 13	100, 8
	1.0		1.0	
Primary patency − at 5 years, %, *n p* value	100, 4	100, 4	100, 8	
			**?**	
Cardiac complications, %, *n p* value	14.3, 3	0	5, 1	11.1, 2
	0.238		0.595	
Respiratory complications, %, *n p* value	38.1, 8	47.1, 8	30, 6	55.6, 10
	0.743		0.188	
Renal complications, % *p* value	9.5, 2	23.5, 4	10, 2	22.2, 4
	0.378		0.395	
Hepatobiliary complications, %, *n p* value	9.5, 2	47.1, 8	5, 1	22.2, 4
	0.743		0.188	
Cerebrovascular complications, %, *n p* value	28.6, 6	11.8, 2	15, 2	27.8, 5
	0.257		0.438	
Bleeding, %, *n p* value	38.1, 8	41.2, 7	25, 5	55.6, 10
	1.0		0.096	
Bleeding required reoperation, %, *n p* value	38.1, 8	41.2, 7	25, 5	55.6, 10
	1.0		0.096	
Peripheral ischemia, %, *n p* value	9.5, 2	0	0	11.1, 2
	0.492		0.218	
Postoperative urinary tract infection, %, *n p* value	14.3, 3	29.4, 5	20, 4	22.2, 4
	0.426		1.0	
Gastrointestinal bleeding, %, *n p* value	4.8, 1	17.6, 3	10, 2	11.1, 2
	0.307		1.0	
Wound infection, %, *n p* value	19, 4	11.8, 2	10, 2	22.2, 4
	0.672		0.383	

**Table 4:** Patients' characteristics noted in previous reports and in the current study [17, 20, 21, 23, 24, 25, 28, 32, 33, 34, 37, 38, 39, 40, 41, 42], in alphabetic order

Author	Sex ratio (m/f)	Mean age, years	Weight loss, *n* (%)	Smoking, *n* (%)	PAD, *n* (%)
Beebe et al. [32]	7/10	54	10/10 (100)	Not reported	Not reported
Calderon et al. [25]	17/20	59	13/20 (65)	6/20 (30)	3/20 (15)
Current study *n* (%)	20/38 (53)	64	32/38 (84)	39/38 (78.9)	23/38 (60.5)
Davenport et al. [37]	119/156	65	54/156 (35)	77/156 (49)	37/156 (24)
Foley et al. [38]	31/49	62	Not reported	48/49 (98)	28/49 (57)
Gentile et al. [20]	16/26	59	Not reported	25/26 (96)	16/26 (62)
Geroulakos et al. [39]	9/10	66	10/10 (100)	Not reported	Not reported
Hollier et al. [34]	11/56	50	55/56 (98)	Not reported	Not reported
Jimenez et al. [24]	33/47	62	39/47 (83)	43/47 (91)	23/47 (49)
Johnston et al. [33]	11/21	58	1/21 (5)	19/21 (90)	17/21 (81)
Kihara et al. [40]	30/42	60	Not reported	37/42 (88)	Not reported
Kruger et al. [41]	22/39	65	37/39 (95)	36/39 (92)	16/39 (41)
Mateo et al. [23]	60/85	62	74/85 (87)	75/85 (88)	Not reported
McMillan et al. [17]	17/25	61	21/25 (84)	22/25 (88)	9/25 (36)
Moawad et al. [28]	19/24	58	14/24 (58)	20/24 (83)	Not reported
Rheudasil et al. [21]	21/41	59	23/41 (56)	36/41 (88)	18/41 (44)
Zelenock et al. [42]	13/23	56	23/23 (100)	Not reported	Not reported

**Table 5:** Postoperative outcome of the current study and of previous reports at the follow-up period [23, 38, 40, 41, 43, 44, 45, 46]

Author	Patients, *n*/vessels, *n*	Technical success (%)	Mortality (%)	Morbidity (%)	Recurrence (%)	1° patency (%)
Cho et al. [43]	25/41	100	0	21	Not reported	57
Current study	38/55	100	13.2	48.4	4.5	81.6
Foley et al. [38]	28/28	100	3	Not reported	10	79
Illuminati et al. [44]	11/12	100	0	27	10	90
Kihara et al. [40]	42/52	100	10	35	10	65
Kruger et al. [41]	39/67	100	2.5	12	5	92
Leke et al. [45]	17/25	100	6	41	0	100
Mateo et al. [23]	85/not reported	100	8	23	20	71
Park et al. [46]	98/179	100	5	21	8	Not reported
