# Supplementary material for: Chronic Mesenteric Ischemia: Differential Vascularsurgical Therapy and Its Outcome in a Single-Center Observational Study
Source: Visc Med. 2021 Nov 29;38(4):255–64. doi: 10.1159/000519423 (PMC9421703; doi:10.1159/000519423)
Supplement: Supplementary file 1 — Supplementary data [file vis-0038-0255-s01.docx]

**Supplementary Table 1:** Pre-, intra- and postoperative risk factors and related clinical outcomes.

| Preoperative risk factors | Intraoperative risk factors | Postoperative risk factors | Clinical outcome |
| --- | --- | --- | --- |
| - Age - Weight loss - Abdominal angina - BMI - Diabetes mellitus - Arterial hypertension - Hyperlipidemia - Smoking history - Preoperative TPN - PAD - CVD - RVD - CHD - CHF - CRF - Previous malignancy - Previous surgeries:   - Abdominal   - Bowel   - Peripheral vascular   - Aortic   - Cardiac   - Mesenteric - Pathology of CA, SMA, and IMA (obstruction / stenosis) - Therapy: antiplatelet/ anticoagulation | - Duration of OP - Duration of aortic cross-clamping - Approach used - Inflow vessel - Outflow vessel(s) - Number of vessels reconstructed - Combination with other constructions - Perioperative antibiotic prophylaxis - Type of material used for the reconstruction | - Length of stay on ICU - Length of stay in the hospital - Reoperation(s)/Re-intervention(s) | 1. **Morbidity**  - Patency (primary and clinical) - Graft obstruction - Graft infection - Intestinal ischemia - Intestinal obstruction - Cardiovascular complications   - Heart failure   - Myocardial  infarction - Respiratory complications - Cerebrovascular complications   - Stroke   - Delirium - Renal failure - Hepatobiliary complications - UTI - Wound infection - Lymphocele - Postoperative bleeding - Gastrointestinal bleeding  1. **Mortality** 2. **Survival rate** |
| BMI: body mass index, TPN: total parenteral nutrition, PAD: peripheral arterial disease, CVD: cerebrovascular disease, RVD: renovascular disease, CHD: coronary heart disease, CHF: congestive chronic heart failure, CRF: chronic renal failure, CA: celiac axis, SMA: superior mesenteric artery, IMA: inferior mesenteric artery, ICU: intensive care unit, UTI: urinary tract infection | | | |
