# Supplementary material for: Chronic Mesenteric Ischemia: Differential Vascularsurgical Therapy and Its Outcome in a Single-Center Observational Study
Source: Visc Med. 2021 Nov 29;38(4):255–64. doi: 10.1159/000519423 (PMC9421703; doi:10.1159/000519423)
Supplement: Supplementary file 2 — Supplementary data [file vis-0038-0255-s02.docx]

**Supplementray Table 2:** Distribution of preoperative risk factors among males and females.

| Risk factor | No. of patients (%) having the risk factor | | | *p* value |
| --- | --- | --- | --- | --- |
|  | **Males** | **Females** | **Total** |  |
| Weight loss | 13 (72.2 %) | 18 (95 %) | 31 (81.6 %) | **0.055** |
| Gastrointestinal bleeding | 2 (11.1 %) | 0 ( 0 %) | 2 ( 5.3 %) | 0.218 |
| Diabetes mellitus | 4 (22.2 %) | 4 (20 %) | 8 (21.1 %) | 0.867 |
| Arterial hypertension | 14 (77.8 %) | 16 (80 %) | 30 (78.9 %) | 0.867 |
| HLP | 8 (44.4 %) | 10 (50 %) | 18 (47.4 %) | 0.732 |
| History of smoking | 18 (100 %) | 17 (85 %) | 35 (92.1 %) | 0.087 |
| Active smoker | 15 (83.3 %) | 15 (75 %) | 30 (78.9 %) | 0.529 |
| PAD | 12 (66.7 %) | 11 (55 %) | 23 (60.5 %) | 0.463 |
| CVD | 8 (44.4 %) | 8 (40 %) | 16 (42.1 %) | 0.782 |
| RVD | 2 (11.1 %) | 1 ( 5 %) | 3 ( 7.8 %) | 0.485 |
| CHD | 7 (38.9 %) | 3 (15 %) | 10 (26.3 %) | **0.095** |
| Atrial fibrillation | 4 (22.2 %) | 1 ( 5 %) | 5 (13.2 %) | 0.117 |
| CHF | 3 (16.7 %) | 2 (10 %) | 5 (13.2 %) | 0.544 |
| CRF | 8 (44.4 %) | 9 (45 %) | 17 (44.7 %) | 0.973 |
| Previous abdominal  surgery | 11 (61.1 %) | 15 (75 %) | 26 (68.4 %) | 0.358 |
| Previous bowel surgery | 10 (55.6 %) | 9 (45 %) | 19 (50.0 %) | 0.516 |
| Previous peripheral  vascular surgery | 5 (27.8 %) | 4 (20 %) | 9 (23.7 %) | 0.573 |
| Previous carotid surgery | 1 ( 5.6 %) | 2 (10 %) | 3 ( 7.9 %) | 0.612 |
| Previous aortic surgery | 5 (27.8 %) | 5 (25 %) | 10 (26.3 %) | 0.846 |
| Previous cardiac surgery | 3 (16.7 %) | 1 ( 5 %) | 4 (10.5 %) | 0.242 |
| Previous mesenteric  surgery | 2 (11.1 %) | 2 (10 %) | 4 (10.5 %) | 0.911 |
| HLP: hyperlipoproteinemia, PAD: peripheral arterial disease, CVD: cerebrovascular disease, RVD: renovascular disease, CHD: coronary heart disease, CHF: congestive heart failure, CRF: chronic renal failure | | | | |
